# Meta-analysis of laparoscopic versus open liver resection for colorectal liver metastases

**DOI:** 10.18632/oncotarget.13026

**Published:** 2016-11-02

**Authors:** Zhi-qiang Tian, Xiao-fang Su, Zhi-yong Lin, Meng-chao Wu, Li-xin Wei, Jia He

**Affiliations:** ^1^ Tumor Immunology and Gene Therapy Center, Eastern Hepatobiliary Surgery Hospital, The Second Military Medical University, Shanghai 200438, China; ^2^ Department of General Surgery, Wuxi People's Hospital Affiliated Nanjing Medical University, Wuxi, Jiangsu 214023, China; ^3^ Department of Rehabilitation and Physiotherapy Medicine, Wuxi Taihu Hospital (101 Hospital of Chinese People's Liberation Army), Wuxi, Jiangsu 214044, China; ^4^ Department of Health Statistics, The Second Military Medical University, Shanghai 200433, China

**Keywords:** colorectal liver metastases, laparoscopic liver resection, open liver resection, meta-analysis

## Abstract

**Background:**

To compare surgical and oncological outcomes of laparoscopic versus open liver resection for colorectal liver metastases.

**Results:**

A total of 14 retrospective studies with 1679 colorectal liver metastases patients were analyzed: 683 patients treated with laparoscopic liver resection and 996 patients with open liver resection. With respect to surgical outcomes, laparoscopic compared with open liver resection was associated with lower blood loss (MD, -216.7, 95% CI, -309.4 to -124.1; *P* < 0.00001), less requiring blood transfusion (OR, 0.36; 95% CI, 0.23 to 0.55; P < 0.00001), lower postoperative complication morbidity (OR, 0.61; 95% CI, 0.47 to 0.80; *P* = 0.003), and shorter hospitalization time (MD, -3.85, 95% CI, -5.00 to -2.71; *P* < 0.00001). However, operation time and postoperative mortality were no significant difference between the two approaches. With respect to oncological outcomes, laparoscopic liver resection group was prone to lower recurrence rate (OR, 0.78; 95% CI, 0.61−0.99; *P* = 0.04), but surgical margins R0, overall survival and disease-free survival were no significant difference.

**Materials and Methods:**

We performed a systematic search in MEDLINE, EMBASE, and CENTRAL for all relevant studies. All statistical analysis was performed using Review Manager version 5.3. Dichotomous data were calculated by odds ratio (OR) and continuous data were calculated by mean difference (MD) with 95% confidence intervals (CI).

**Conclusions:**

Laparoscopic and open liver resection for colorectal liver metastases have the same effect on oncological outcomes, but laparoscopic liver resection achieves better surgical outcomes.

## INTRODUCTION

Colorectal cancer is the third most common malignancies, and the liver is the most frequent site of metastasis [1, 2]. Approximately 50% of colorectal cancer patients occur liver metastasis during disease evolution, which is a major cause of cancer death [1, 3]. Liver resection is the only potential curative treatment for colorectal liver metastases with studies reporting 5-year survival rates of approximately 35–60% [3–7].

Laparoscopic surgery and open laparotomy are two surgical approaches of liver cancer resection. In recent years, laparoscopic liver resection is a growing option in the field of liver cancer surgery. Multiple studies [8–11] have attested to the effective in surgical outcomes and long-term oncologic outcomes of laparoscopic liver resection. However, no randomized controlled trial has been completed comparing laparoscopic with open liver resection for colorectal liver metastases [12]. Further, the related evidence of laparoscopic liver resection for colorectal liver metastases have not been systematically reviewed.

Although several meta-analyses [13–15] of observational studies have evaluated short- and long-term outcomes for laparoscopic liver resection, but there are limited data. It is necessary to carry out an updated meta-analysis in accordance to the Preferred Reporting Items for Systematic Reviews and Meta-Analyses (PRISMA) Statement [16] by pooling data from all of the available studies. Thus, we conduct a meta-analysis to identify and screen the benefits of laparoscopic compared with open liver resection for treatment colorectal liver metastases.

## RESULTS

### Selected studies

**Figure 1:**
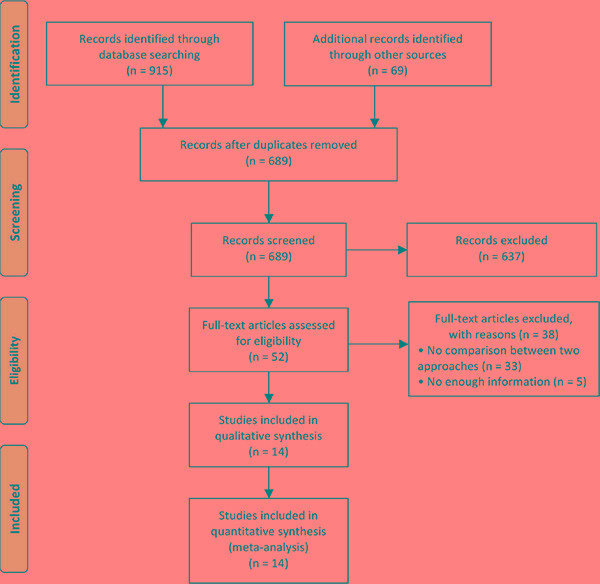
Flow diagram showing selection of relevant articles in the meta-analysis

Our initial search strategy yielded 689 potential articles after removing duplicates in a combined search of MEDLINE (PubMed), EMBASE and CENTRAL databases covering studies published until October 18th 2016 and a manual approach. 637 articles were excluded on the basis of their titles and abstracts according to the inclusion and exclusion criteria. A full-text examination of the remaining 52 articles was performed. 38 additional articles were further ruled out for the reasons outlined in Figure 1. In the end, 14 studies published between 2002 and 2015 were included in quantitative synthesis in this meta-analysis. A flow diagram of the further details on selection process was shown in Figure 1.

### Study characteristics

All included 14 studies [17–30] were retrospective studies that were conducted in Belgium (1), China (2), France (2), Japan (5), Norway (1), UK (1), and US (2). The informations about surgeons involved in these studies were not reported. 7 studies [17–19, 21, 25, 27, 28] had sample sizes greater than 100 patients. The baseline characteristics of these 14 studies in the meta-analysis were summarized in Table 1. A total of 1679 colorectal liver metastases patients were included in the meta-analysis: 683 patients treated with laparoscopic liver resection and 996 patients with open liver resection. All of studies stated that participators were consecutively enrolled.

**Table 1:** Baseline characteristics of the studies included in the meta-analysis

Study (author, years, country)	Design	Arms	No. of patients	Age (years)	Male (%)	Tumor size (cm)	No. of Tumors	MH (*N*)	Conversion (*n*, %)	Follow-up (median, m)
Abu et al., 2014, UK	retrospective	LLR	55	66 (42–85)	51	3.15 (0.3–9)	1 (1–3)	19	6 (12)	22
		OLR	85	67 (47–86)	65	NA	NA	46		28
Bepuu et al., 2015, Japan	PSM analysis	LLR	171	> 70 (*n* = 61)	63	> 5 (*n* = 4)	> 2 (*n* = 44)	NR	NA	> 60^†^
		OLR	342	> 70 (*n* = 103)	63	> 5 (*n* = 8)	> 2 (*n* = 91)	NR		> 60^†^
Cannon et al., 2012, US	retrospective	LLR	35	62 ± 10	NA	4 ± 3	1 ± 1	19	NA	> 60^†^
		OLR	138	62 ± 11	NA	5 ± 3	1 ± 1	71		> 60^†^
Castaing et al., 2009, France	retrospective	LLR	60	62 ± 11	62	3.3 ± 1.1	2.2 ± 2.3	26	6 (10)	30
		OLR	60	62 ± 11	62	4.4 ± 3.8	2.2 ± 2.0	24		33
Cheung et al., 2012, China	retrospective	LLR	20	58 (42–74)	65	1.5 (0.5–4.5)	1 (1–2)	1	0	NA
		OLR	40	64 (29–83)	73	2.2 (0.5–7)	1 (1–2)	2		NA
de'Angelis et al., 2015, France	PSM analysis	LLR	52	63 (32–81)	52	2.6 (1.5–6)	1 (1–4)	18	3 (5.8)	59
		OLR	52	63 (46–83)	56	3.0 (1.5–5.2)	1 (1–5)	22		54
Guerron et al., 2013, US	retrospective	LLR	40	66 ± 1.9	53	3.3 ± 0.3	1.3 ± 0.1	5	2 (5)	16
		OLR	40	62 ± 1.8	38	3.2 ± 0.3	1.7 ± 0.1	9		16
Hasegawa et al., 2015, Japan	retrospective	LLR	100	67 (24–91)	64	2.3 (0.7–9.5)	1 (1–8)	20	1 (1)	29
		OLR	68	65 (37–83)	43	3.5 (1.1–16)	2 (1–12)	25		36
Inoue et al., 2013, Japan	retrospective	LLR	23	66 ± 9.6	48	2.5 ± 1.1	≥ 2 (*n* = 0)	4	1 (4)	NA
		OLR	24	68 ± 9.5	54	2.7 ± 0.9	≥ 2 (*n* = 3)	5		NA
Iwahashi et al., 2013, Japan	retrospective	LLR	21	68 ± 11	76	2.4 ± 0.8	1.9 ± 1.1	4	NA	> 60^†^
		OLR	21	68 ± 10	67	2.6 ± 0.8	2.1 ± 1.2	5		> 60^†^
Kubota et al., 2014, Japan	retrospective	LLR	43	64 ± 11	51	≥ 5(*n* = 8)	≥ 2 (*n* = 16)	7	NA	37
		OLR	62	66 ± 12	65	≥ 5 (*n* = 20)	≥ 2 (*n* = 39)	25		37
Mala et al., 2002, Norway	retrospective	LLR	13	68 (55–73)	31	2.6 (1–6)	2 (1–7)	2	0	NA
		OLR	14	50 (24–74)	29	3 (1.5–9)	1 (1–4)	2		NA
Qiu et al., 2013, China	retrospective	LLR	30	53 ± 12	47	2.5 ± 2.0	≥ 2 (*n* = 10)	2	2 (6.6)	NA
		OLR	30	NA	50	2.8 ± 1.5	≥ 2 (*n* = 9)	5		NA
Topal et al., 2012, Belgium	retrospective	LLR	20	57.6	50	4 (0.4–7)	2 (1–6)	20	NA	43.4
		OLR	20	66.0	40	3.291–12.5	2 (1–14)	20		43.4

PSM, propensity score matching; LLR, laparoscopic liver resection; OLR, open liver resection; MH, major hepatectomy (≥ 3 segments of the liver); NA, not available; No., number; m, month;

† upper ends of follow-up range.

### Quality judgments of studies

The quality judgment for each study was performed with Newcastle-Ottawa Scale (NOS) [31]. The evaluation stars of each study based on NOS judgment were shown in Table 2. All studies reviewed consecutive colorectal liver metastases patients in the selection of patients. However, laparoscopic surgery was manipulated in selected patients who were suitable, and the patients accepting open surgery were selectively matched. All included studies were comparable. The mean value for the 14 retrospective cohort studies assessed was 6.9 stars. Overall, all of included studies were evaluated as being moderate to high quality.

**Table 2:** Quality assessment of studies in the meta-analysis based on newcastle-ottawa scale

Study (Author, years)	Selection				Comparability	Outcome			Quality judgment
	1	2	3	4	1	1	2	3	
Abu et al., 2014	★	-	★	★	★	★	-	★	★★★★★★
Bepuu et al., 2015	★	-	★	★	★★	★	★	★	★★★★★★★★
Cannon et al., 2012	★	★	-	-	★★	★	★	★	★★★★★★★
Castaing et al., 2009	★	★	★	-	★★	★	-	★	★★★★★★★
Cheung et al., 2012	★	★	★	★	★	★	-	★	★★★★★★★
de'Angelis et al., 2015	★	-	★	-	★★	★	★	★	★★★★★★★
Guerron et al., 2013	★	★	★	★	★	★	-	★	★★★★★★★
Hasegawa et al., 2015	★	★	★	-	★★	★	★	★	★★★★★★★★
Inoue et al., 2013	★	-	★	-	★★	★	-	★	★★★★★★
Iwahashi et al., 2013	★	★	-	-	★★	★	★	★	★★★★★★★
Kubota et al., 2014	★	★	★	-	★	★	★	★	★★★★★★★
Mala et al., 2002	★	-	-	-	★★	★	-	★	★★★★★
Qiu et al., 2013	★	★	★	★	★★	★	-	★	★★★★★★★★
Topal et al., 2012	★	★	-	-	★★	★	-	★	★★★★★★

### Meta-analysis of surgical outcomes

With respect to surgical related outcomes, six endpoints including operation time, blood loss, perioperative blood transfusion, postoperative complication morbidity, postoperative mortality, and hospitalization time were taken into analysis. Dichotomous data was calculated by odds ratio (OR) and continuous data was calculated by mean difference (MD) with 95% confidence intervals (CI).

### Operation time

**Figure 2:**
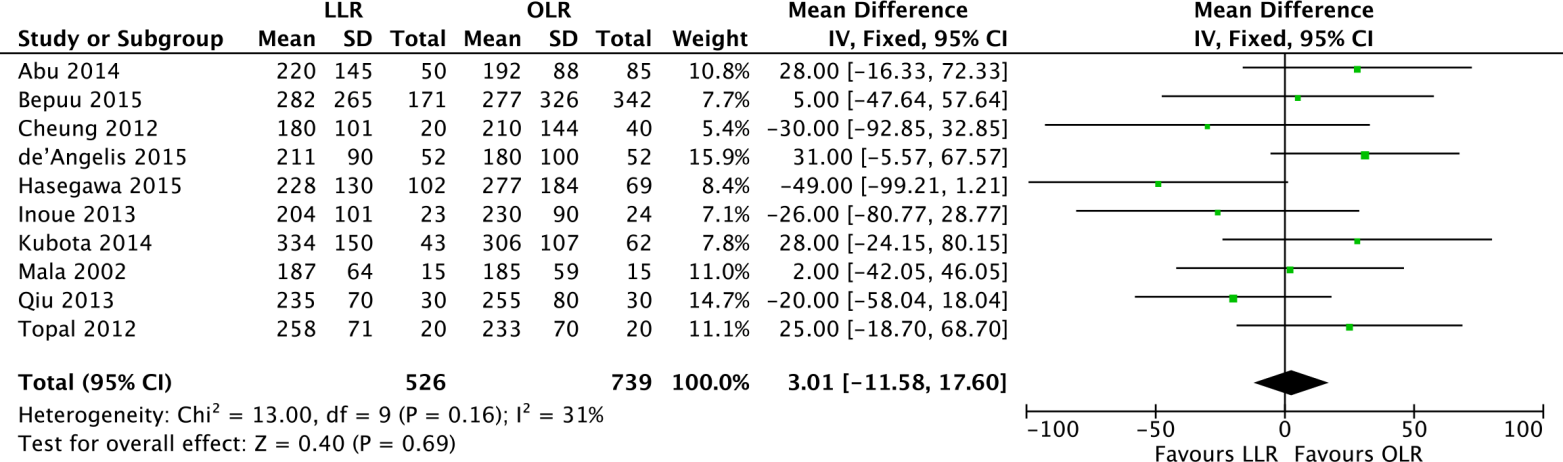
Forest plot displaying operation time (min) of the meta-analysis LLR, laparoscopic liver resection; OLR, open liver resection.

The operation time was available from 10 studies [17, 18, 20, 21, 23, 25, 26, 28–30]. Analysis indicated that there was low heterogeneity among the studies (*P* = 0.16, *I^2^* = 31%) and a fixed effect model was used. Based on the complete analysis, operation time was assessed with no significant difference between laparoscopic and open liver resection for colorectal liver metastases (MD, 3.01; 95% CI, -11.6 to 17.6; *P* = 0.69). (Figure 2).

### Blood loss

**Figure 3:**
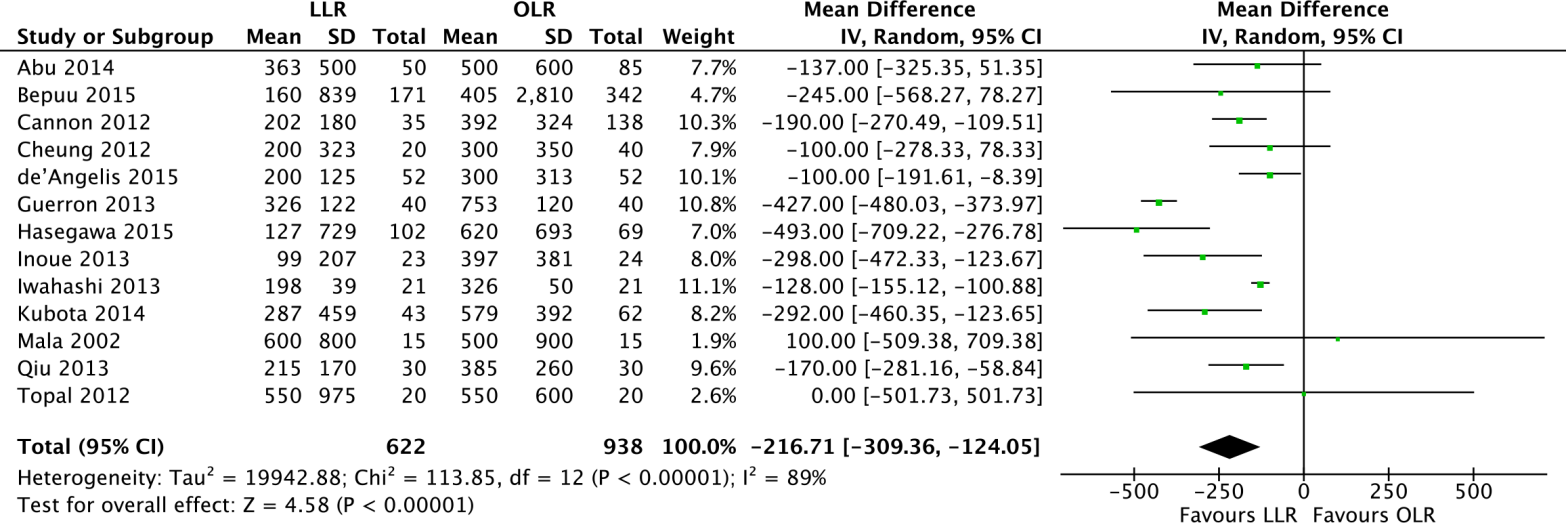
Forest plot displaying blood loss (ml) of the meta-analysis LLR, laparoscopic liver resection; OLR, open liver resection.

Mean changes of blood loss were pooled for the 13 studies [17, 18, 20–30]. Heterogeneity was high (*P* < 0.00001, *I^2^* = 89%) and a random effect model was used. Intra-operative blood loss was significantly lower in laparoscopic liver resection than in open liver resection (MD, -216.7, 95% CI, -309.4 to -124.1; *P* < 0.00001). (Figure 3).

### Perioperative blood transfusion

**Figure 4:**
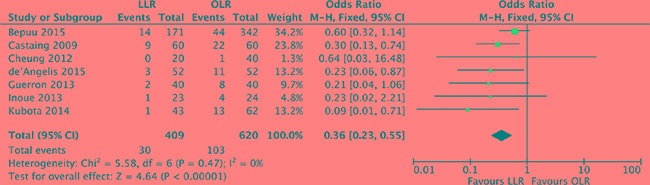
Forest plot displaying perioperative blood transfusion of the meta-analysis LLR, laparoscopic liver resection; OLR, open liver resection.

The relative risk of perioperative blood transfusion was available form 7 studies [18–23, 25]. Analysis indicated that there was no heterogeneity among the studies (*P* = 0.47, *I^2^* = 0%) and a fixed effect model was used. The proportion of patients requiring blood transfusion was lower in laparoscopic liver resection than in open liver resection (OR, 0.36; 95% CI, 0.23 to 0.55; *P* < 0.00001). (Figure 4).

### Postoperative complication morbidity

**Figure 5:**
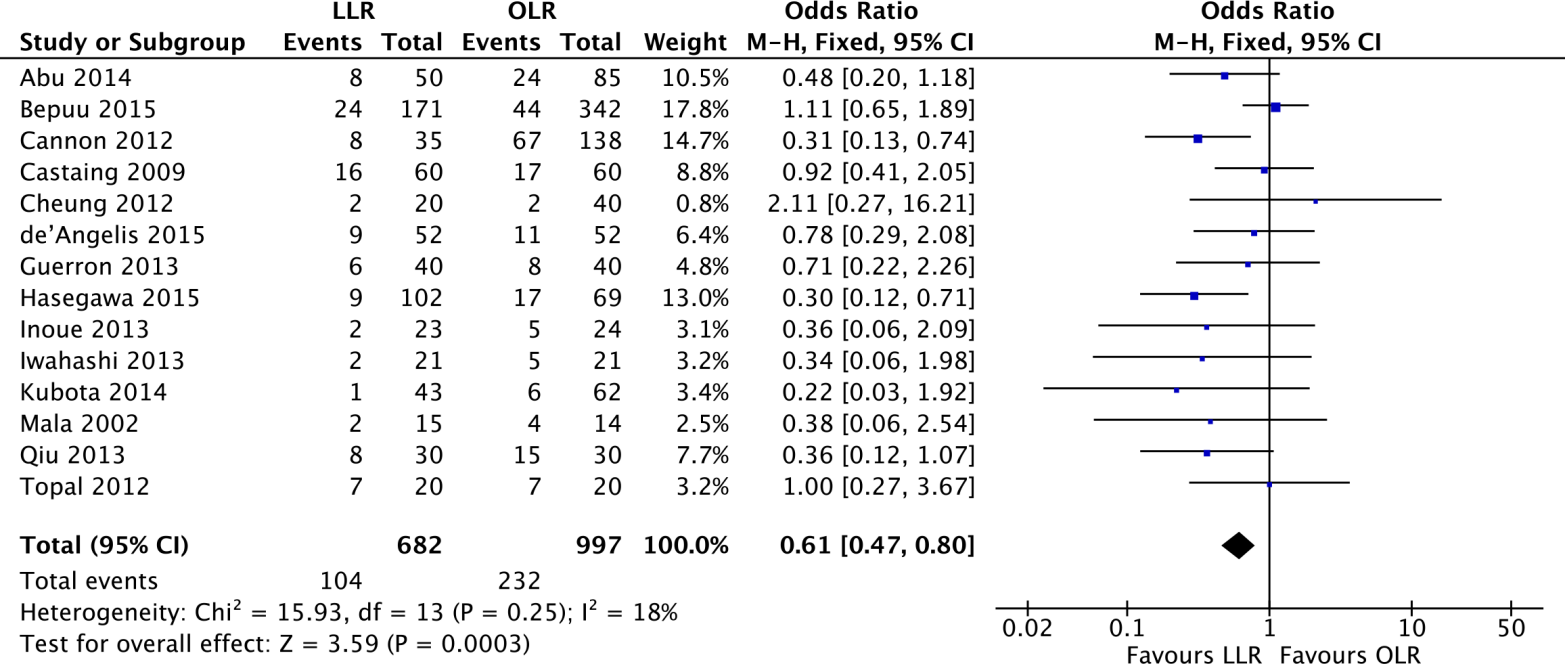
Forest plot displaying postoperative complication morbidity of the meta-analysis LLR, laparoscopic liver resection; OLR, open liver resection.

All of 14 studies [17–30] reported on the postoperative complication morbidity rate. There was low significant heterogeneity among the studies (*P* = 0.25, *I^2^* = 18%) and a fixed effect model was used. Patients in the laparoscopic liver resection had lower morbidity than in open liver resection (OR, 0.61; 95% CI, 0.47 to 0.80; *P* = 0.0003). (Figure 5).

### Postoperative mortality

**Figure 6:**
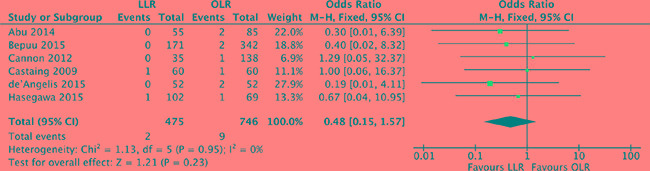
Forest plot displaying postoperative mortality of the meta-analysis LLR, laparoscopic liver resection; OLR, open liver resection.

The relative risk of postoperative mortality was available for 6 studies [17–19, 21, 27, 28]. There was no heterogeneity among the studies (*P* = 0.95, *I^2^* = 0%) and a fixed effect model was used. Perioperative mortality did not differ significantly between laparoscopic and open liver resection for colorectal liver metastases (OR, 0.48; 95% CI, 0.15 to 1.57; *P* = 0.23). (Figure 6).

### Hospitalization time

**Figure 7:**
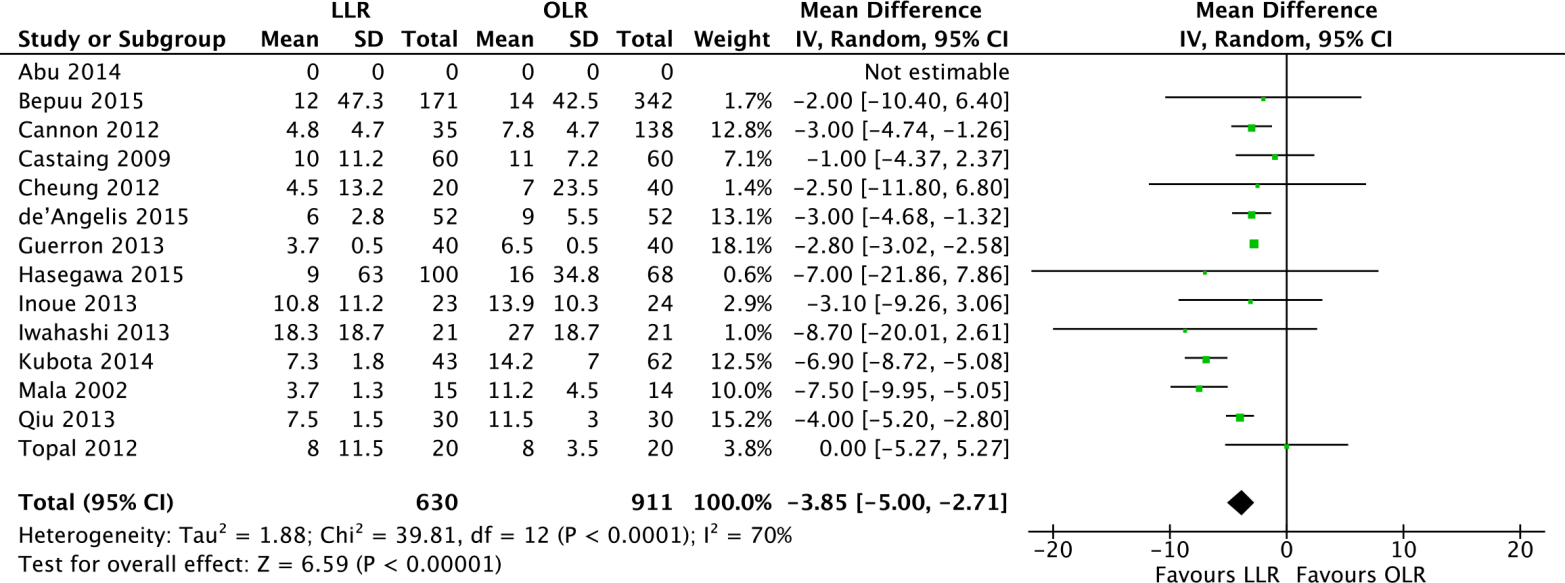
Forest plot displaying hospitalization time (days) of the meta-analysis LLR, laparoscopic liver resection; OLR, open liver resection.

The length of hospitalization time was pooled for all of 14 studies [17–30]. Heterogeneity was high among the studies (*P* < 0.0001, *I^2^* = 70%) and a random effect model was used. The pooled analysis showed that hospitalization time of laparoscopic liver resection was shorter than of open liver resection (MD, -3.85 days, 95% CI, -5.00 to -2.71; *P* < 0.00001). (Figure 7).

### Meta-analysis of oncological outcomes

With respect to oncological related outcomes, four endpoints including surgical margins R0, recurrence, disease-free survival, and overall survival were taken into analysis.

### Surgical margins R0

**Figure 8:**
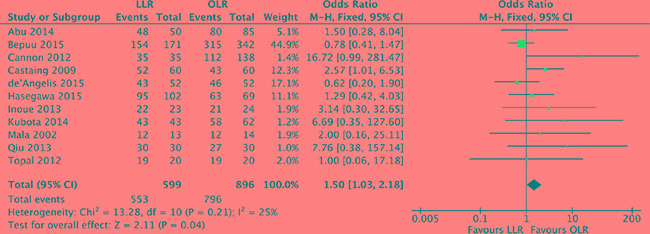
Forest plot displaying surgical margins R0 of the meta-analysis LLR, laparoscopic liver resection; OLR, open liver resection.

The pathological resection margin status was reported in 11 studies [17–19, 21, 23, 25–30]. Heterogeneity was low (*P* = 0.21, *I^2^* = 25%) and a fixed effect model was used. The pooled analysis showed that surgical margins R0 was assessed with difference in laparoscopic liver resection in comparison of OLR (OR, 1.50; 95% CI, 1.03 to 2.18; *P* = 0.04). Open liver resection was prone to higher surgical margins R0, but the difference was slight. (Figure 8).

### Recurrence

**Figure 9:**
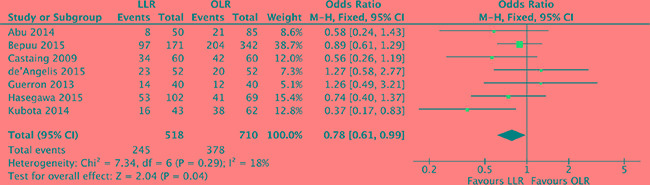
Forest plot displaying recurrence of the meta-analysis LLR, laparoscopic liver resection; OLR, open liver resection.

Concerning local recurrence, we pooled all hepatic and extra-hepatic recurrence of the included studies. 7 studies [17–19, 21, 22, 25, 28] were hit into our analysis with low heterogeneity (*P* = 0.29, *I^2^* = 18%) and a fixed effect model was used. Our outcome was prone to a lower recurrence rate in laparoscopic approach compared with open liver resection (OR, 0.78; 95% CI, 0.61–0.99; *P* = 0.04). (Figure 9).

### Disease-free survival

**Figure 10:**
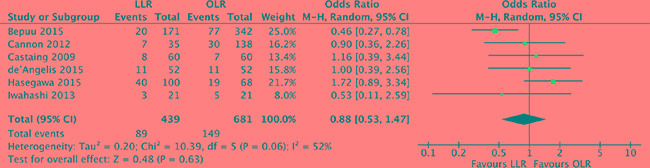
Forest plot displaying disease-free survival of the meta-analysis LLR, laparoscopic liver resection; OLR, open liver resection.

The 5-year disease-free survival was available form 6 studies [18, 19, 21, 24, 27, 28]. Heterogeneity was high (*P* = 0.06, *I^2^* = 52%) and a random effect model was used. The 5-year disease-free survival was no significant difference between laparoscopic and open liver resection for colorectal liver metastases (OR, 0.88; 95% CI, 0.53 to 1.47; *P* = 0.63). (Figure 10).

### Overall survival

**Figure 11:**
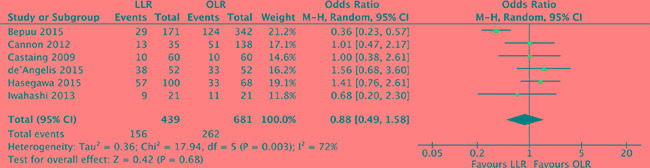
Forest plot displaying overall survival of the meta-analysis LLR, laparoscopic liver resection; OLR, open liver resection.

There were 6 studies [18, 19, 21, 24, 27, 28] reported the 5-year overall survival, which was the crucial endpoint. Heterogeneity was high (*P* = 0.003, *I^2^* = 72%) and a random effect model was used. The findings indicated that the 5-year overall survival was no significant difference between laparoscopic and open liver resection for colorectal liver metastases (OR, 0.88; 95% CI, 0.49 to 1.58; *P* = 0.68). (Figure 11).

### Publication bias

**Figure 12:**
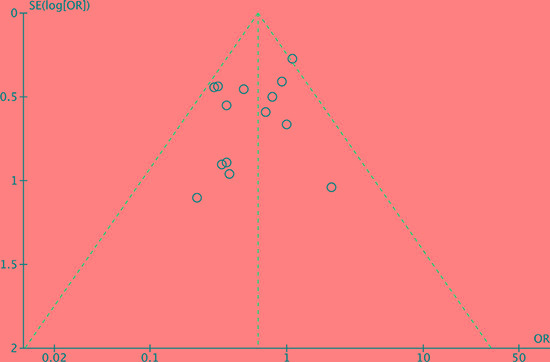
Funnel plot of postoperative complication morbidity analysis of publication bias

The funnel plot of postoperative complication morbidity were assessed to evaluate the reliability of publication bias in this meta-analysis [32]. The funnel plot was basically inverted and funnel-shaped with bilateral symmetry, indicating that there was no publication bias. (Figure 12).

## DISCUSSION

Laparoscopic liver resection for colorectal liver metastases has not been widely accepted [15]. The mainly reason is lack of convincing evidence in adequacy comparison of surgical outcomes and oncological value [14]. To date, numerous retrospective and comparative studies [17–30] have reported the feasibility, safety, and efficacy of laparoscopic liver resection for colorectal liver metastases. However, no randomized controlled trial has been completed on laparoscopic compared with open liver resection for colorectal liver metastases for ethical and other reasons. As far as we know, one randomized controlled trial (the Oslo-CoMet Study, NCT01516710) [12] is in progress comparing laparoscopic with open liver resection for colorectal liver metastases, but the long-term result will not be available before 2020. Therefore, non-randomized studies may thus be useful by providing information on treatment effects. Our goal of this meta-analysis was to evaluate the surgical and oncological outcomes of laparoscopic compared with open liver resection for colorectal liver metastases. So, we comprehensively identified all relevant non-randomized studies and summarized the evidence on surgical and oncological outcomes.

With respect to surgical outcomes, postoperative complication morbidity is a major cause of patient suffering and societal costs. Our analysis demonstrates that laparoscopic liver resection acquired lower postoperative morbidity than open liver resection. Similar finding is also reported by multiple observational studies, in which morbidity rate for laparoscopic liver resection varies from 11% to 15% [33–35] and for open liver resection varies from 22% to 47% [36, 37]. In terms of the other surgical outcomes, our analysis showed that colorectal liver metastases patients with laparoscopic liver resection acquired lower blood loss, less blood transfusion requirements and shorter hospitalization time than with open liver resection. However, postoperative mortality was no significantly different between the two approaches. This meta-analysis demonstrates that laparoscopic liver resection for colorectal liver metastases achieves better surgical outcomes than open liver resection.

The oncological outcomes are the core indicators for resection treatment of colorectal liver metastases. Specific concerns about the oncologic adequacy of laparoscopic liver resections include port site metastases, the trophic effect of pneumoperitoneum on malignant cells, and the surgical margins R0 [38]. In this meta-analysis, none of the 14 included studies identified port site metastases. Oncological of R0 resection is the gold standard of care of colorectal liver metastases. In this study, surgical margins R0 is assessed with no significant difference in laparoscopic liver resection compared with open liver resection. Instead, laparoscopic liver resection is prone to a lower recurrence rate than open liver resection. More importantly, the pooled analysis shows that 5-year disease-free survival and overall survival are not significant difference between laparoscopic and open liver resection. Therefore, laparoscopic liver resection has no compromises effect on oncologic outcomes compared with open liver resection for colorectal liver metastases.

There are several limitations in this meta-analysis. First, the included studies were mainly retrospective comparative studies with inherent limitations related to the significant risk of selection [39] and reporting bias [40]. The overall quality of evidence for all pooled results was relatively low. Second, colorectal liver metastases patients treated with laparoscopic liver resections were a highly selected population. Laparoscopic liver resection requires extensive experience and expertise than open liver resection and is associated with a learning curve. Finally, most of included studies were relatively small sample sizes and lack of long-term oncological data. Only 6 studies had follow-up longer than 5 years. Additionally, many of the studies were from single a single site, which may affect generalizability. Larger and well definitive randomized controlled trials would increase this study's predictive strength.

## CONCLUSIONS

Our meta-analysis summarizes the best available evidence for surgical and oncological results in directly comparative research studies of laparoscopic compared with open liver resection for colorectal liver metastases. This study demonstrates that laparoscopic liver resection is associated with lower blood loss, less requiring blood transfusion, lower postoperative complication morbidity, shorter hospitalization time, and lower recurrence rate. These findings provide evidence to support laparoscopic liver resection is efficacious for colorectal liver metastases. However, results are limited to none of randomized controlled trials and lack data about the long-term oncological results. The evidences call for larger, well design, and long-term outcomes studies to fully characterize the efficacy of laparoscopic liver resections for colorectal liver metastases.

## MATERIALS AND METHODS

### Study selection and search strategy

A comprehensive search was performed in MEDLINE (PubMed), EMBASE and CENTRAL databases to identify all relevant studies available from their inception to October 18th 2016. We also searched trial registries of ongoing trials. When the criteria for inclusion or exclusion of a study were controversial, the corresponding author was consulted.

The search strategy followed the identification and screening guidelines established by PRISMA statement. The following Mesh search headings and key words were used: (“laparoscopic liver resection” or “laparoscopic hepatectomy”) and (“colorectal cancer” or “colorectal liver metastases”). These terms were used in different Boolean combinations. Our search was restricted to full-length articles published in English. We retrieved all eligible studies and evaluated the reference lists of the identified studies and reviews.

### Inclusion criteria

We included the following studies from the meta-analysis: (1) study design: comparing laparoscopic with open liver resection for colorectal liver metastases patients, (2) include more than 10 patients in each group (minimum of 20 patients), (3) the studies provided surgical and oncologic outcomes, and (4) available data for each surgical regimen. The most recent was used if dual (or multiple) studies were reported by the same institution. Study designs included randomized controlled trials and retrospective/prospective cohort or case-control studies.

### Exclusion criteria

The exclusion criteria of the meta-analysis were the following: (1) studies not reporting clinical outcomes of effectiveness, (2) just one surgical regimen (laparoscopic or open) was reported, (3) other minimally invasive surgery such as radiofrequency ablation was compared, and (4) abstracts, letters, editorials and expert opinions, reviews without original data, case reports.

### Data extraction and outcomes of interest

Data was independently extracted and the quality of included studies were screened by two review authors (T.Z. and X.S.). The two authors extracted the interest variable data of included studies and entered into a dedicated database. The following data of each study was extracted: study characteristics (first author, study designs, surgical approaches, numbers of patients, and follow-up time), demographic measures (publication year, country, age, and gender), surgical outcomes (operation time, blood loss, perioperative blood transfusion, postoperative complication morbidity, postoperative mortality, and hospitalization time), and oncologic outcomes (surgical margins R0, recurrence, overall survival, disease-free survival). We defined perioperative mortality as 90-day hospital death, and hospitalization time including postoperative time and total hospital time. Local recurrence which were observed till the end of follow-up included hepatic only, extra-hepatic only, and both hepatic and extra-hepatic. Hepatic only and extra-hepatic complications were both contained in complications. The data accuracy and completeness were checked by two other authors (J.H. and L.W.). Discrepancies were resolved by consensus in all authors.

### Risk of bias assessment

The risk of bias of the cohort studies was assessed using the Newcastle-Ottawa Scale [31]. The Newcastle-Ottawa Scale star system ranged 0 to 9 stars: 4 stars for selection, 2 stars for comparability, and 3 stars for outcome. In this mate-analysis, a study of the final score > 6 star was regard as a high-quality study. Risk of bias of the included studies were independently assessed by two review authors (T.Z. and X.S.). Disagreement was resolved by consensus in all authors.

### Data synthesis and statistical analysis

This study was statistical analyzed by using Review Manager version 5.3.5 [41]. Most of included studies are retrospective cohort studies. So, OR is used to calculate the dichotomous data in this meta-analysis. The continuous data were calculated by MD with 95% CI. We derived the missing standard deviations from other statistics, such as *P* values or CI if needed. For example, *P* = 0.00001 was assumed when a *P* value was reported as *P* < 0.00001. Cochran's *Q* test and the degree of inconsistency (*I^2^*) were used to assess heterogeneity among combined study results. A fixed-effects model was used if a *P* > 0.05 and *I^2^* < 50%. Otherwise, data were pooled by using the random-effects. *P* < 0.05 indicated statistical significance in the integration results. Publication bias in outcomes was assessed and treated using standard methodology. The funnel plots were used to analyze publication bias.
